# Follicle stimulating hormone increases spermatogonial stem cell colonization during *in vitro* co-culture 

**Published:** 2013

**Authors:** Reza Narenji Sani, Parviz Tajik, Mohammad Hassan Yousefi, Mansoureh Movahedin, Babak Qasemi-Panahi, Shiva Shafiei, Mahmood Ahmadi Hamedani

**Affiliations:** 1 *Department of Theriogenology, Faculty of Veterinary Medicine, University of Tehran, Tehran, Iran; *; 2 *Faculty of Veterinary Medicine, Semnan University, Semnan, Iran; *; 3 *Department of Anatomical Sciences, School of Medical Sciences, Tarbiat Modarres University, Tehran, Iran; *; 4 *Department of Animal Science, Faculty of Agriculture, University of Tabriz, Tabriz, Iran.*

**Keywords:** Bovine, Co-culture, FSH, Sertoli, SSCs

## Abstract

The complex process of spermatogenesis is regulated by various factors. Studies on spermatogonial stem cells (SCCs) have provided very important tool to improve herd genetic and different field. 0.2 to 0.3 percent of total cells of seminiferous tubules is consist of spermatogonial stem cells. To investigate and biomanipulation of these cells, proliferation and viability rate of cells should be increased *in vitro*, at first. Follicle stimulating hormone (FSH) has been suggested to play a determinant role in the survival of germ cells in addition to increasing spermatogonial proliferation. In this study, the *in vitro* effects of FSH on spermatogonial cell colony formation were investigated. Sertoli and spermatogonial cells were isolated from 3-5 months old calves. The identity of the Sertoli cells and spermatogonial stem cells were confirmed through immunocytochemistry and colony morphology, respectively. Co-cultured Sertoli and spermatogonial cells were treated with FSH in different dose of 10, 20 and 40 IU mL^-1^ FSH, before colony assay. Results indicated that, FSH increased *in vitro *colonization of spermatogonial cells in comparison with control group. In conclusion, using FSH provided proper bovine spermatogonial stem cell culture medium for *in vitro *study of these cells.

## Introduction

Spermatogenesis is a complex developmental process that originates from spermatogonial stem cell (SSC). This process consists of sequential, highly organized steps of cell proliferation and differentiation resulting in generation of functional spermatozoa.^1^ Many growth factors, hormones and cell interactions of germ cells with Sertoli cells regulate these processes, and the failure of any of these processes lead to male infertility.^2^

In rodents, the A single (As) spermatogonia are considered the stem cells of spermatogenesis.^3^^-^^5^ Upon division of the As spermatogonia, the daughter cells either separate each other and become two new stem cells, or stay together through an intercellular bridge and become A-paired (Apr) spermatogonia. The Apr spermatogonia develop into chains of four, eight or 16 A-aligned (Aal) spermatogonia. The Aal spermatogonia differentiate into A1 spermatogonia and after six mitotic divisions differentiated to A2, A3, A4 and B spermatogonia, which give rise to spermatocytes at the last mitotic division. Regarding species-specific differences, in bovine testis, a comparable classification with another terminology has been reported.^6^ In this species, spermatogonial precursor cells have been divided into basal stem cells, aggregated spermatogonial precursor cells and committed spermatogonial precursor cells. According to this classification, As-Apr spermatogonia, Aal spermatogonia and A1-A4 differentiating spermatogonia are the result of spermatogonia stem cell differentiation, respectively. Hence, in bulls, Apr spermatogonia are also thought to have stem cell properties.^6^

Adult mammalian testis, has multiple generations of germinal cells, therefore purification of spermatogonia is more difficult than before puberty. Bellve *et al.* obtained 90% pure fraction of type A spermatogonia from immature mice.^7^ Izadyar *et al*. concluded that when testis from 5-month-old calves were used, approximately 1 × 10^6 ^type A spermatogonia per gram of testis with purity about 75% could be obtained routinely.^8^

Sertoli cells have vital roles in testis spermatogenic function for many reasons.^9^ These somatic cells generate and maintain the cytoarchitecture of germinal epithelium, produce nutrients that provide energy substrates to the germ cells and in primates, represent the only cellular component of blood–testis barrier.^10^ The tyrosine kinase c-kit receptor is expressed in germ cells and its ligand, stem cell factor (SCF), is expressed in Sertoli cells of the testis. The interaction between c-kit and SCF is essential for the maintenance and/or mitosis of differentiating type-A spermatogonia.^11^ Co-cultures of gonocytes and Sertoli cells have been used to demonstrate the survival effects of added growth factors on germ cells.^12^

Among the all hormones implicated in spermatogenesis, FSH has been suggested to play a determinant role in the survival of germ cells in addition to increasing spermatogonia proliferation.^13^ The binding of FSH to its receptor has been reported to result in dissociation of the alpha-subunit of receptor-associated G protein, which in turn, leads to activation of adenylyl cyclase and production of cyclic adenosine monophosphate (cAMP).^14^ The catalytic subunit of protein kinase A is released by cAMP, allowing for phosphorylation of numerous intracellular proteins including transcriptional activator and cAMP response element binding protein. Studies on several nonprimate species have demonstrated that FSH binding in testis is restricted to Sertoli cells.^14^ Follicle stimulating hormone (10-1000 ng mL^-1^) causes an increase in intracellular Ca^2+^ within seconds of stimulation.^15^^-^^18^ Reportedly, it act in adult rats by either stimulating germ cell development and/or inhibiting germ-cell degeneration.^19^ The addition of FSH to Sertoli cell culture has been reported to increase the secretion of the mitogenic factor.^20^ This hormone may be quantitatively required for maintenance of spermatogenesis in adult beagles.^21^ To date, attempts to *in vitro *culture of spermatogonia have unsuccessful results in purifying the testicular cell population and also lack of knowledge about the *in vitro *regulation of proliferation and differentiation.^22^ Difficulty of co-culture system is resulted from complex interaction between germ cells and somatic cells and lack of demonstrable *in vitro* cell division.

In studies about spermatogonial isolation and purification, colony morphology is one of the methods in spermatogonial stem cell identification.^8^ Consequently, the aim of the present study was to determine the effects of FSH on spermatogonial cell colony formation after *in vitro *co-culture with Sertoli cells.

## Materials and Methods

Testicular biopsies were obtained from 3 to 5 months old calves undergoing testicular sperm extraction (TESE) procedure. Obtained testis pieces were mechanically minced and floated in Dulbecco's modiﬁed Eagle's medium (DMEM; Sigma Chemical Co., St. Louis, Mo. USA) containing 1 mg mL^-1 ^collagenase, 1 mg mL^-1 ^Trypsin, 1 mg mL^-1 ^hyaluronidase type II and 5 µg mL^-1 ^DNase I and then incubated at 37 ˚C for 60 min.^23^ After three times washing in DMEM and excluding the interstitial cells, for secondary digestion step, seminiferous tubules were incubated in DMEM containing collagenase, hyaluronidase and DNase for 45 min. Finally, obtained cellular suspension was centrifuged at 30 *g* for 2 min to achieve favorite cell population. Then, spermatogonial cells were co-cultured with Sertoli cells for 13 days. For Sertoli cell collection, 5 µg mL^-1 ^Datura stramonium agglutinin-lectin (DSA; Sigma Chemical Co., St. Louis, Mo. USA) in phosphate-buffered saline was poured into the sterile flasks. Cells obtained from secondary enzymatic digestion was added to DSA-lectin coated flasks and incubated at 37 ˚C for 1 hr. After Sertoli cells confluency, spermatogonial cells co-cultured in seven groups, for 13 days.

For co-culture of these cells, DMEM with 10% fetal bovine serum (FBS; Sigma Chemical Co., St. Louis, Mo. USA) 100 ng mL^-1^ Glial cell line-derived neurotrophic factor (GDNF; Sigma Chemical Co., St. Louis, Mo. USA), 100 IU mL^-1^ penicillin and 100 mg mL^-1 ^streptomycin were used. Our experimental groups included Control, group 1 (10 IU mL^-1 ^FSH), group 2 (20 IU mL^-1 ^FSH), group 3 (40 IU mL^-1 ^FSH). Culture medium plus mentioned doses of FSH were refreshed every 3 days. Cell viability was evaluated by means of the dye exclusion test (0.04% trypan blue solution).

**Cells identification. **For Sertoli cells identification, we used Vimentin immunocytochemical staining (Sigma Chemical Co., St. Louis, Mo. USA) which was described by Anway *et al.* and Tajik *et al.* Also, Colony morphology of SSCs was used for SSCs identification. ^24^^,^^25^

**Colony assay. **Number and surface area of spermatogonial cell derived colonies were measured on days 4, 7, 10 and 13. For the measurements we used inverted microscope (Olympus, Tokyo, Japan) equipped with ocular grid. 

**Statistical analysis. **The data was analyzed using SPSS for Windows (Version 16, IBM Corporation, Somers, NY, USA). Results are expressed as mean ± SD. The statistical significance between mean values was determined by one-way ANOVA and Duncan post hoc test; *p <*0*.*05 was considered significant.

## Results

**Fig. 1:**
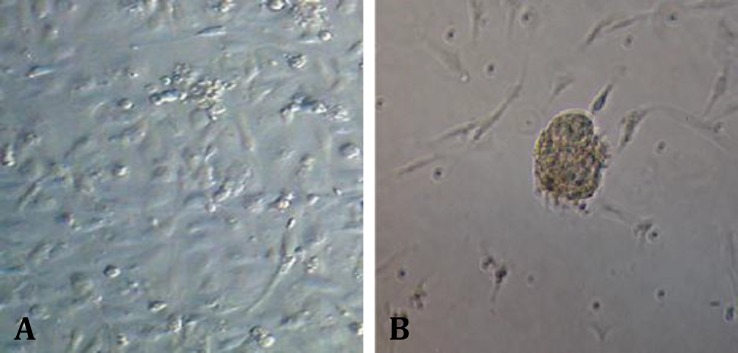
**A.** Sertoli cells that created a monolayer of cells. **B.** The morphology of a spermatogonial derived colony that formed from co-cultured spermatogonial cells on a monolayer of Sertoli cells, 200×.

**Fig. 2:**
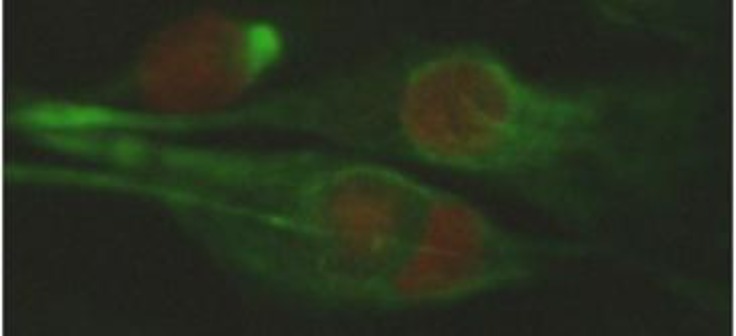
Immunocytochemical staining of bovine Sertoli cell with anti-vimentin conjugated with fluorescein isothiocyanate, 1000×.

**Isolation and identification of spermatogonial and Sertoli cells. **The cell population obtained from seminiferous tubules of 3-5 months old calves testis contained mostly two cell types with different morphology characteristics. The first cell type were proliferated and created a monolayer of cells (Fig. 1A), whereas the other cell type created a colony after proliferation has been made. These colonies have morphology characteristics of bovine SSCs colonies; they were round, with distinct margin and with brown color that were placed on Sertoli cells layer (Fig. 1B). Moreover, Vimentin, which is a molecular marker for Sertoli cells, was detected in the feeder monolayer cells (Fig. 2).

**Comparison among experimental groups. **Number of colonies was significantly higher than control group after the 7^th^ day of co-culture regardless of FSH doses used (*p* ≤ 0.05) (Table 1). Also, until the 10^th^ day of co-culture the surface area of colonies were similar with ones in control group, but in the 10^th^ day, the surface area of colonies were smaller than ones in control group and after that, almost reached the surface area of colonies in control group (*p* ≤ 0.05) (Table 2).

The surface area of colonies in control group was similar in FSH group (Table 2). There was not any significant difference to appearance of colonies in all groups. All groups showed increasing of colony number until the 10^th^ day of co-culture and after that decreasing of one, but just in FSH groups this increase were significant. Morphology of colony was similar between all groups. There was no significant difference in viability rate between all groups and these rates were more than 85% in all of these at the end of co-culture (Table 3).

**Table 1:** Comparison of colony numbers between control and experimental groups at different times. Data are presented as mean ± SD.

**Groups**	**Day 4**	**Day 7**	**Day 10**	**Day 13**
**Control**	14.00 ± 3.74	19.60 ± 6.65 b	21.20 ± 6.38 b	20.60 ± 7.09 b
**FSH (10 IU L** ^-1^ **)**	23.20 ± 15.40	62.00 ± 26.76 a	71.62 ± 7.78 a	55.20 ± 23.41 a
**FSH (20 IU L** ^-1^ **)**	31.20 ± 26.18	53.20 ± 27.92 a	71.40 ± 22.43 a	55.20 ± 21.06 a
**FSH (40 IU L** ^-1^ **)**	30.40 ± 25.20	55.20 ± 31.58 a	75.20 ± 17.71 a	56.00 ± 32.55 a

ab The value with different letter significantly differ within column (*p <*0*.*05).

**Table 2:** Comparison of colony surface area (mm^2^) between control and experimental groups at different times. Data are presented as mean ± SD.

**Groups**	**Day 4**	**Day 7**	**Day 10**	**Day13**
**Control**	135.72 ± 87.04	202.88 ± 15.22	195.08 ± 18.46 b	163.52 ± 83.28
**FSH (10 IU L** ^-1^ **)**	69.84 ± 65.11	94.10 ± 10.06	66.00 ± 51.90 a	68.74 ± 14.39
**FSH (20 IU L** ^-1^ **)**	69.60 ± 54.37	101.74 ± 13.46	47.14 ± 6.46 a	68.26 ± 13.10
**FSH (40 IU L** ^-1^ **)**	71.40 ± 68.49	95.34 ± 11.07	52.40 ± 19.10 a	86.08 ± 21.34

ab The value with different letter significantly differ within column (*p <*0*.*05).

**Table 3:** Comparison of viability rate between control and experimental groups at the end of co-culture

**Groups**	**Viability rate**
**Control**	85.70%
**FSH (10 IU L** ^-1^ **)**	95.60%			
**FSH (20 IU L** ^-1^ **)**	85.40%			
**FSH (40 IU L** ^-1^ **)**	95.10%			

There is no significant difference between groups.

## Discussion

Suitable populations of Sertoli cells and SSCs could be obtained from 3 to 5 months old calves, because the seminiferous epithelium of calves’ testis contains two distinct cell types: type-A SSCs and Sertoli cells. It appears that, 3-5 months, is most appropriate age of calves for type A SSCs isolation. Most of tubule cross-sections contained type A SSCs, therefore this testis was proved to be the best source for isolation of this type of SSCs. Highly enriched populations of type A SSCs with final purity of up to 75% could be isolated routinely. Cell recovery was about 1 × 10^6^ type A, SSCs per gram of testis and the viability of isolated SSCs was always > 80%.^8^ Our viability rate results are comparable to those reported for the isolation of type A SSCs from prepubertal mice, rats, pigs and bovine.^7^^,^^8^^,^^26^^,^^27^

About Sertoli cells, immature ones proliferate, so the final number of Sertoli cells is determined before adulthood. It appears that there are fundamental differences between species as to when Sertoli cells proliferate, but in bull, there is a short gap (weeks) between the neonatal and peripubertal periods.^28^ Also recent study showed that there is proliferation in Sertoli cells after maturation.^25^

Therefore, the optimum time for recovery of Sertoli cells and primitive type A SSCs is 5^th^ month after birth.^8^ Therefore, we used 3-5 months old calves as model animal in our study. 

Koruji *et al.* concluded that some SSCs co-cultures (e.g. co-culture with Sertoli cells and growth factors) had considerable effect on colonization of SSCs, supported SSCs survival and proliferation.^29^ Therefore, co-culture of SSCs with Sertoli cells had supportive effect on proliferation of SSCs. Hence, we did co-culture of SSCs and Sertoli cells in this project. 

In this research, isolated cells from the seminiferous tubules of 3-5 months old calves had two types of cells with distinct immunocytochemical feature, similar to Sertoli cells and type-A SSCs. These findings are in agreement with those were reported by Koruji *et al.* who demonstrated the immunocytochemical features of Sertoli cells and SSCs in mouse.^29^

Specific marker for detection of sertoli cells was Vimentin immunocytochemistry staining.^24^^,^^25^ Colonies had bovine SSCs morphology.^30^ Therefore, we assume that the colonies may have been largely derived from the SSCs.

Our approach was *in vitro* co-culture of Sertoli cells and SSCs and determined the effects of FSH on the colonization of SSCs. Proper factor was chosen for proliferation increscent of co-cultured SSCs. In FSH-treated groups, surface area of colonies was smaller than control group until 10^th^ day of co-culture but just in the 10^th^ day, this difference was significant. These results were similar with Anjamrooz *et al*.^31^ Also number of colonies in these groups was higher than control group.

Results of Anjamrooz *et al*. were contrary to our results.^31^ Hence, proliferative SSCs can create colonies and choose the proliferative pathway. This pathway could have been induced by Sertoli cell- secreted growth factors. Follicle stimulating hormone appears to stimulate Sertoli- proliferation both *in vivo* and *in vitro*;^32^ it is the major mitotic factor for Sertoli cells.^33^ FSH stimulate Sertoli cells to secret Sertoli-cell-secreted growth factors; which (these factors) stimulate the proliferation of type-A SSCs.^34^

In conclusion we postulated that FSH increased *in vitro* co-culture colony number of SSCs. Thus it could be chosen as a factor for *in vitro* SSCs study, also according to its price in comparison with other factors, maybe it was the best factor for *in vitro* SSCs study. 
